# Recompensation following first decompensation in patients with alcohol-related cirrhosis

**DOI:** 10.1186/s12876-026-04651-6

**Published:** 2026-02-02

**Authors:** Ji Yoon Kwak, Hankyu Jeon, Hyeon Uk Kwon, Jae Eun Kim, Ji Hee Han, Jung Woo Choi, Ra Ri Cha, Jae Min Lee, Sang Soo Lee

**Affiliations:** 1 Department of Internal Medicine, Gyeongsang National University School of Medicine and Gyeongsang National University Changwon Hospital, 11, Samjeongja-ro, Seongsan-gu, Changwon, Gyeongnam 51472 Republic Of Korea; 2 Institute of Medical Sciences, Gyeongsang National University, Jinju, Republic Of Korea; 3 Department of Internal Medicine, Gyeongsang National University School of Medicine and Gyeongsang National University Hospital, Jinju, Korea, Republic of (South Korea)

**Keywords:** Alcohol abstinence, Alcohol-related cirrhosis, Recompensation, Further decompensation, Baveno VII consensus

## Abstract

**Background:**

The Baveno VII consensus recently introduced the term “recompensated cirrhosis,” but its prognostic relevance in alcohol-related cirrhosis remains unclear. This study aims to analyze the frequency and associated factors of recompensation in patients with alcohol-related cirrhosis following the first decompensation and evaluate the prognosis based on recompensation status.

**Methods:**

This prospective cohort study was conducted at a tertiary care hospital in South Korea between 2017 and 2022. We enrolled 184 consecutive patients with alcohol-related cirrhosis presenting with a first decompensating event. According to the Baveno VII criteria, recompensation was defined as alcohol abstinence, improvement in liver function, and resolution of ascites/encephalopathy (off diuretics/lactulose) and absence of recurrent variceal bleeding (for at least 12 months). Patients were followed for outcomes including further decompensation, liver transplantation, or death.

**Results:**

Of the 184 patients, 45 (24.5%) achieved abstinence-induced recompensation while 38 (20.6%) died during the index hospitalization. In the multivariable Fine-Gray competing risk regression analysis, a lower baseline Child-Pugh score (sHR = 0.77, 95% CI = 0.61–0.98, *P* = 0.032) and lower MELD score (sHR = 0.93, 95% CI = 0.88–0.99, *P* = 0.023) were independent factors associated with recompensation. Among the 146 survivors, 51 (34.9%) died, 10 (6.8%) underwent liver transplantation, and 95 (65.1%) experienced further decompensation. Time-dependent multivariable Cox regression analysis showed that recompensation decreased the risk of death or liver transplantation by 96% (HR = 0.04, 95% CI = 0.01–0.30, *P* = 0.002) and the risk of further decompensation by 75% (HR = 0.25, 95% CI = 0.13–0.48, *P* < 0.001).

**Conclusions:**

Approximately one-fourth of the patients with alcohol-related cirrhosis achieved recompensation after first decompensation, which was strongly associated with reduced mortality and lower risk of further decompensation.

## Introduction

Globally, alcohol-associated liver disease (ALD) is a leading cause of liver-related mortality and accounts for approximately half of all deaths from liver cirrhosis [1]. Alcohol-related cirrhosis remains one of the most common indications for liver transplantation (LT) [2]. The natural course of cirrhosis is typically divided into two distinct phases: compensated and decompensated. While compensated cirrhosis is often asymptomatic, decompensated cirrhosis is characterized by complications associated with portal hypertension, including ascites, hepatic encephalopathy (HE), and variceal bleeding (VB) [3]. Notably, many individuals with alcohol-related cirrhosis already present with signs of decompensation at the time of diagnosis [4–6].

Traditionally, the transition from compensated to decompensated cirrhosis has been regarded as irreversible [7]. However, the Baveno VII consensus introduced the concept of recompensation, defined as at least partial regression of the structural and functional derangements of cirrhosis after removal of the underlying cause [8]. In ALD, alcohol abstinence constitutes the removal of the primary etiology. It is well established that abstinence not only improves long-term survival but is also linked to the amelioration of portal hypertension, which is the main driver of decompensation [9]. Successful management of the primary etiology, such as a sustained virologic response in hepatitis C, durable viral suppression in hepatitis B, and alcohol abstinence in alcohol-related cirrhosis, can result in recompensation. Most studies utilizing the Baveno VII criteria for recompensation have focused on hepatitis B or C [10–15], with reported recompensation rates of 19–56% for hepatitis B and 25%–63% for hepatitis C. However, Hofter et al. reported that, according to the Baveno VII criteria, recompensation was achieved in 18% of 204 abstinent patients with alcohol-related cirrhosis [16].

Further decompensation is associated with a higher mortality rate than the first decompensation [17]. Further decompensation, according to the Baveno VII criteria, is identified by any of the following events: (1) the manifestation of a second decompensating event resulting from portal hypertension (ascites, HE, or VB) and (2) the emergence of recurrent VB, recurrent ascites, recurrent HE, development of spontaneous bacterial peritonitis (SBP), and/or hepatorenal syndrome-acute kidney injury (HRS-AKI) [8].

However, few studies have investigated the clinical course of patients with alcohol-related cirrhosis after the first decompensating event, particularly regarding recompensation, further decompensation, LT, and death. Therefore, this study aimed to investigate the overall clinical course of patients with alcohol-related cirrhosis following their first decompensating event. Specifically, we analyzed the incidence and predictors of recompensation according to the Baveno VII criteria and evaluated the long-term prognosis based on recompensation status.

## Methods

### Study population

In this single-center prospective observational study, we considered all consecutive patients with alcohol-related cirrhosis who presented to Changwon Gyeongsang National University Hospital between January 2017 and December 2022. The study included patients who met the following criteria: (1) age > 20 years, (2) diagnosis of alcohol-related cirrhosis, (3) presence of ascites, HE, and/or VB as the first decompensating event, and (4) absence of other etiologies, including hepatitis B, hepatitis C, autoimmune hepatitis, and Wilson’s disease. Patients were excluded if they met any of the following criteria: (1) prior diagnosis of hepatocellular carcinoma (HCC), (2) loss to follow-up within 12 months, (3) presence of severe extrahepatic disease or other malignancy, or (4) presence of portal vein thrombosis, prior transjugular intrahepatic portosystemic shunt insertion, or prior LT. The diagnosis of alcohol-related cirrhosis was based on clinical signs of portal hypertension and radiological findings accompanied by thrombocytopenia. Alcohol-related cirrhosis was diagnosed in patients with a history of excessive alcohol consumption (defined as an average daily intake > 40 g for males and 20 g for females) in the absence of other identifiable causes of liver disease [18]. This study was conducted in accordance with the 1964 Declaration of Helsinki and approved by the Institutional Review Board of Gyeongsang National University Changwon Hospital (GNUCH 2017-07-003). All participants provided informed consent prior to enrollment.

### Definitions

First decompensation was defined as the occurrence of overt ascites (grade ≥ 2), VB, or overt HE (West Haven grade ≥ 2) [19, 20]. According to the Baveno VII criteria, hepatic recompensation required all of the following: 1) removal of the underlying etiology (i.e. abstinence from alcohol); 2) resolution of ascites (without diuretics), HE (without lactulose/rifaximin), and VB, with no recurrence for at least 12 months (the date of resolution was defined as the date of the clinical visit when the patient remained free of symptoms after discontinuing specific medical therapies); and 3) sustained improvement in liver function, indicated by a Model for End-Stage Liver Disease (MELD) score < 10 and/or a Child-Pugh class A. Further decompensation, as defined by the Baveno VII criteria, was defined as: 1) the development of a second decompensating event due to portal hypertension (ascites, HE, or VB); 2) recurrence or worsening of VB, ascites, HE; and 3) development of SBP and/or HRS-AKI. Alcohol abstinence was defined as no alcohol consumption for at least 6 months following the first decompensating event. This 6-month threshold was selected based on the widely accepted ‘6-month rule’ for LT candidacy, which serves as a surrogate for sustained abstinence and low risk of relapse [21].

### Follow-up and data collection

The index date (Day 1) was the date of initial hospitalization due to the first decompensation. All patients were active drinkers at the time of the index decompensation. Patients were followed up until death, LT, or study end (December 31, 2024). Baseline data collected included age, sex, body mass index, type of first decompensating event, mean arterial pressure, presence of acute-on-chronic liver failure (ACLF) based on the EASL-CLIF consortium criteria [22], Child-Pugh class, MELD score, and alcohol consumption. Laboratory assessments included hemoglobin, platelet count, prothrombin time-international normalized ratio (PT-INR), bilirubin, aspartate aminotransferase, alanine aminotransferase, albumin, creatinine, and sodium.

During follow-up, all participants underwent clinical evaluations, laboratory tests, and imaging every 3–6 months, or more frequently as needed. At each visit, the following information was collected from each patient: (1) cirrhosis-related complications, LT, HCC, and death; and (2) alcohol consumption, assessed through interviews with patients and their relatives.

### Statistical analysis

Categorical data are presented as frequencies and percentages. Continuous variables are expressed as medians with interquartile ranges. The Mann–Whitney U test was used to compare continuous variables. Categorical variables were compared using the Pearson’s chi-square test or Fisher’s exact test, as appropriate.

Factors associated with recompensation were identified using Fine-Gray competing risk regression models, considering death and LT as competing events. Variables with a *P* value < 0.05 in univariable analysis were included in the multivariable analysis. Results from the Fine-Gray models are presented as subdistribution hazard ratios (sHRs) with 95% confidence intervals (CIs).

Time-dependent univariable and multivariable Cox regression models were used to analyze the impact of recompensation on outcomes, including death or LT. According to the Baveno VII criteria, recompensation can only be defined after a sustained 12-month period after decompensation. Therefore, recompensation was treated as a time-dependent covariate, and all patients were classified as non-recompensated at baseline and reclassified upon meeting recompensation criteria. Once patients met the criteria for recompensation, they retained this classification in the survival analysis regardless of subsequent alcohol relapse, capturing the real-world clinical trajectory following the achievement of recompensation. A similar time-dependent Cox regression model was used to evaluate the association between recompensation and the risk of further decompensation. Results from the Cox regression models are presented as HRs with 95% CIs.

A Sankey diagram was used to illustrate the clinical course following the first decompensating event, emphasizing the different clinical trajectories based on recompensation and further decompensation status. All statistical analyses were performed using the IBM SPSS Statistics version 24 (IBM Corp., Armonk, NY, USA) and R 4.2.2 statistical software (R Core Team, R Foundation for Statistical Computing, Vienna, Austria).

## Results

### Patient characteristics

In this cohort of 184 patients with alcohol-related cirrhosis following their first decompensating event, the median follow-up duration was 29.3 months. The median age was 55.5 years, and 82.1% were male (Table 1.). The most common decompensating events were ascites (70.7%), HE (24.5%), and VB (29.9%). Twenty-seven patients experienced more than one decompensating event. At enrollment, 63 patients (34.2%) developed ACLF during hospitalization for the first decompensation. The median MELD score was 18.5, with 86 patients (46.6%) in Child-Pugh class B and 92 (50%) in class C.

**Table 1:** Patients characteristics at the first decompensation (*N* = 184)

	Total (N=184)	No recompensation (N=139)	Recompensation (N=45)	*P*
Age, year	55.5 (48.0 - 62.0)	54.0 (48.0 - 62.0)	57.0 (50.0 - 62.0)	0.253
Male gender	151 (82.1%)	113 (81.3%)	38 (84.4%)	0.823
*BMI,*kg/m*^2^	22.5 (19.8 - 25.4)	22.6 (19.6 - 25.4)	22.1 (20.3 - 23.2)	0.547
First decompensating events				
Ascites	130 (70.7%)	102 (73.4%)	28 (62.2%)	0.187
Hepatic encephalopathy	45 (24.5%)	39 (28.1%)	6 (13.3%)	0.048
Variceal bleeding	55 (29.9%)	40 (28.8%)	15 (33.3%)	0.578
≥2 first decompensating events	27 (18.5%)	23 (22.8%)	4 (8.9%)	0.064
Death at the first decompensation	38 (20.7%)	38 (27.3%)	0 (0%)	<0.001
Jaundice	110 (59.8%)	94 (67.6%)	16 (35.6%)	<0.001
MAP, *mmHg *	103.0 (87.0 - 117.0)	103.0 (87.0 - 117.0)	103.0 (88.0 - 122.0)	0.454
ACLF at first decompensation	63 (34.2%)	57 (90.5%)	6 (9.5%)	<0.001
Child-Pugh class (A/B/C)	3.3%/46.7%/50.0%	16.7%/67.4%/87.0%	83.3%/32.6%/13.0%	<0.001
MELD score	18.5 (13.0 - 26.0)	21.0 (14.0 - 29.0)	13.0 (11.0 - 17.0)	<0.001
Hemoglobin, *g/dL*	9.9 (8.0 - 12.2)	9.7 (7.7 - 12.3)	10.6 (8.5 - 11.8)	0.465
Platelet,×10^9^/L	106.5 (66.0 - 155.5)	101 (66 - 147.0)	129.0 (66.0 - 164.0)	0.297
PT-INR	1.49 (1.29 - 1.98)	1.60 (1.36 - 2.23)	1.28 (1.15 - 1.50)	<0.001
Bilirubin, *mg/dL*	4.1 (2.0 - 8.5)	4.6 (2.4 - 9.5)	2.9 (2.7 - 3.3)	<0.001
AST, *U/L*	106.0 (56.0 - 206.0)	106.0 (57.0 - 208.0)	97.0 (52.5 - 206.0)	0.856
ALT, *U/L*	34.0 (19.0 - 73.8)	33.0 (18.0 - 67.0)	44.0 (23.5- 89.5)	0.105
Albumin, *g/dL*	2.7 (2.4 - 3.0)	2.7 (2.2 - 3.0)	2.9 (2.7 - 3.3)	<0.001
Creatinine,* mg/dL *	0.91 (0.69 - 1.84)	0.94 (0.70 - 2.25)	0.83 (0.63 - 1.46)	0.124
Sodium, *mmol/L*	134.0 (128.9 - 137.1)	133.8 (128.1 - 136.6)	134.5 (131.5 - 139.0)	0.042

*BMI* Body mass index, *ICU* Intensive care unit, *MAP* Mean arterial pressure, *ACLF* acute-on-chronic liver failure, *MELD* Model for End-Stage Liver Disease, *PT-INR* Prothrombin time- international normalized ratio, *AST* Aspartate aminotransferase, *ALT* Alanine aminotransferase

Data are presented as the medians (interquartile range) for continuous data and percentages for categorical data

*Data are missing for some patients

### Hepatic recompensation

During the study period, 45 patients (24.5%) achieved recompensation. The cumulative incidence rates of recompensation at 1, 2, and 3 years were 32.8%, 36.6%, and 37.8%, respectively, with most recompensations occurring approximately 12 months after the first decompensation. There were no significant differences in age, sex, body mass index, or mean arterial pressure between patients with and without recompensation (Table 1.). The frequencies of encephalopathy, jaundice, Child-Pugh class C, and ACLF were lower in patients with recompensation than in those without. In addition, the MELD scores, PT-INR, and bilirubin levels were lower in patients with recompensation than in those without recompensation. Conversely, the serum albumin and sodium levels were higher in patients who achieved recompensation than in those who did not. These findings suggest that better-preserved liver function at the time of the first decompensating event may be predictive of subsequent recompensation.

### Factors associated with recompensation

In the univariable analysis, factors significantly associated with recompensation included having absence of ACLF, lower Child-Pugh score, and a lower MELD score (all *P* < 0.05; Table 2.). In the multivariable analysis, both the Child-Pugh score (sHR = 0.77, 95% CI = 0.61–0.98, *P* = 0.032) and MELD score (sHR = 0.93, 95% CI = 0.88–0.99, *P* = 0.023) remained independent predictors of recompensation.

**Table 2:** Fine-Gray competing risk regression analysis for factors associated with recompensation at baseline (N=184)

Variable	Univariable analysis		Multivariable analysis	
	*P*	sHR (95% CI)	*P*	sHR (95% CI)
No ACLF	0.004	3.42 (1.48 – 7.88)	0.490	0.70 (0.25 – 1.93)
Child-Pugh score (per point)	<0.001	0.66 (0.57 - 0.77)	0.032	0.77 (0.61 – 0.98)
MELD score (per point)	<0.001	0.90 (0.86 - 0.95)	0.023	0.93 (0.88 – 0.99)

*sHR* subdistribution hazard ratio, *CI* Confidence interval, *ACLF* Acute-on-chronic liver failure, *MELD* Model for End-Stage Liver Disease

### Liver transplantation or death

Among the total cohort, 89 patients died and 10 underwent LT during the study period. The majority of deaths (85/89, 95.5%) were liver-related, primarily due to ACLF, septic shock, and gastrointestinal bleeding. The cumulative incidence of death or LT at 1, 2, 3, and 5 years were 33.7%, 42.9%, 49.6%, and 57.1%, respectively. Notably, 38 patients (20.7%) died during hospitalization for the first decompensation. Among the 146 patients who survived the index hospitalization, 72 (49.3%) achieved alcohol abstinence, and 47 (32.2%) maintained abstinence until the end of the study. These 146 survivors were followed for a median duration of 37.3 months to evaluate long-term clinical outcomes, such as death, LT, and further decompensation (Table 3.). Among them, 51 (34.9%) died and 10 (6.8%) underwent LT. Remarkably, the rate of death or LT was significantly lower in patients with recompensation than in those without (1/45 [2.2%] vs. 60/101 [59.4%], *P* < 0.001), highlighting the substantial clinical benefit of recompensation. However, recompensation did not appear to affect the risk of HCC development.

**Table 3:** Characteristics of further decompensation based on recompensation achievement (*N *= 146)

	Total (*N*=146)	No recompensation (*N*=101)	Recompensation (*N*=45)	*P*
Alcohol abstinence	72 (39.1%)	27 (19.4%)	45 (100%)	<0.001
Persistent alcohol abstinence	47 (32.2%)	14 (13.9%)	33 (73.3%)	<0.001
Death/LT	61 (41.8%)	60 (59.4%)	1 (2.2%)	<0.001
Death	51 (34.9%)	50 (49.5%)	1 (2.2%)	<0.001
LT	10 (6.8%)	10 (100%)	0 (0%)	0.032
HCC	8 (5.5%)	5 (5.0%)	3 (6.7%)	0.702
No further decompensation	51 (34.9%)	17 (16.8%)	34 (75.6%)	<0.001
Further decompensating events	95 (65.1%)	84 (83.2%)	11 (24.4%)	
Uncomplicated ascites	35 (24.0%)	29 (28.7%)	6 (13.3%)	0.058
SBP	6 (4.1%)	6 (5.9%)	0 (0%)	0.177
HRS-AKI	21 (14.4%)	20 (19.8%)	1 (2.2%)	0.004
HE	21 (14.4%)	21 (20.8%)	0	<0.001
VB	28 (19.2%)	23 (22.8%)	5 (11.1%)	0.115

*LT* Liver transplantation, *HCC* Hepatocellular carcinoma, *SBP* Spontaneous bacterial peritonitis, *HRS-AKI* Hepatorenal syndrome–acute kidney injury, *HE* Hepatic encephalopathy, *VB* Variceal bleeding

### Factors associated with liver transplantation or death

In the univariable analysis of the 146 patients who survived at first decompensation, a high Child-Pugh score, high MELD score, absence of recompensation, low hemoglobin level, and low sodium level were associated with death or LT (Table 4.). In the multivariable time-dependent Cox regression analysis, recompensation (adjusted HR = 0.04, 95% CI = 0.01–0.30, *P* = 0.002) and hemoglobin level (adjusted HR per g/dL = 0.85, 95% CI = 0.77–0.94, *P* = 0.001) remained independently associated with a lower risk of death or LT.

**Table 4:** Time-dependent Cox regression analysis for factors associated with liver transplantation or death in patients who survived at first decompensation (*N*=146)

Variable	Univariable analysis		Multivariable analysis	
	*P*	HR (95% CI)	*P*	HR (95% CI)
Recompensation	0.001	0.04 (0.01 - 0.28)	0.002	0.04 (0.01 - 0.30)
Child-Pugh score (per point)*	0.006	1.22 (1.06 - 1.41)	0.444	1.07 (0.89 - 1.29)
MELD score (per point)*	0.003	1.05 (1.02 - 1.09)	0.360	1.02 (0.98 - 1.07)
Hemoglobin (per *g/dL*)*	0.005	0.87 (0.80 - 0.96)	0.001	0.85 (0.77 - 0.94)
Sodium (per *mmol/L*)*	0.022	0.95 (0.92 - 0.99)	0.132	0.96 (0.92 - 1.01)

*HR* Hazard ratio, *CI* Confidence interval, *MELD* Model for End-Stage Liver Disease

* Baseline laboratory values at first decompensation

### Further decompensation

Among 146 patients who survived the first decompensation, 95 (65.1%) experienced further decompensation (Table 3.). The further decompensating events included uncomplicated ascites (35, 24.0%), SBP (6, 4.1%), HRS-AKI (21, 14.4%), HE (21, 14.4%), and VB (28, 19.2%). Notably, further decompensation occurred significantly less frequently in patients who achieved recompensation than in those who did not (11/45 [24.4%] vs. 84/101 [83.2%], *P* < 0.001), indicating a strong protective effect of recompensation. We further analyzed the 11 recompensated patients who experienced a subsequent event. Among them, 7 patients had relapsed to alcohol use, and the majority (5/7) presented with recurrent ascites. In contrast, 4 patients maintained abstinence but developed further decompensation, primarily manifesting as variceal bleeding (3/4). This suggests that while abstinence effectively resolves ascites, the risk of variceal bleeding due to residual portal hypertension may persist. Conversely, among the 51 patients who did not experience further decompensation, only two (3.9%) died or underwent LT, compared to 59 (62.1%) of the 95 patients who did (*P* < 0.001).

### Factors associated with further decompensation

**Fig. 1:**
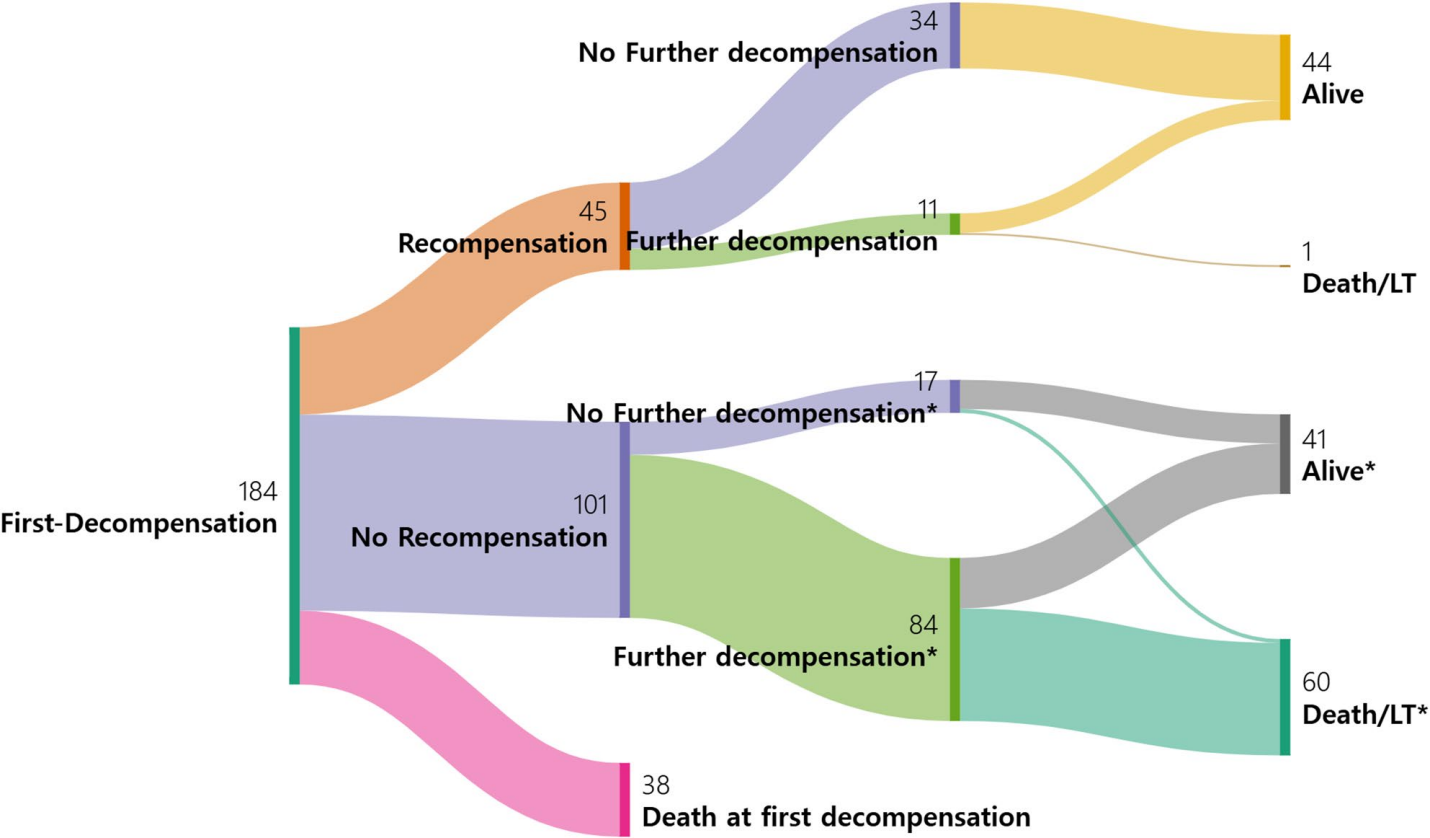
Clinical course of patients with alcoholic cirrhosis following the first decompensating events

In the univariable analysis, the absence of recompensation, high MELD score, high PT-INR, low albumin level, and low hemoglobin level were associated with further decompensation (Table 5.). In the multivariable time-dependent Cox regression analysis, the following were identified as independent predictors: recompensation (adjusted HR = 0.25, 95% CI = 0.13–0.48, *P* < 0.001), PT-INR (adjusted HR per unit = 2.42, 95% CI = 1.48–3.95, *P* < 0.001), and hemoglobin level (adjusted HR per g/dL = 0.92, 95% CI = 0.85–0.99, *P* = 0.029). These findings highlight the strong predictive value of recompensation in preventing further decompensation events. The clinical course of patients with alcohol-related cirrhosis following the first decompensation, stratified by recompensation and further decompensation status, is illustrated in a Sankey diagram (Fig. 1), illustrating the transitions between different clinical states.

**Table 5:** Time-dependent Cox regression analysis for factors associated with further decompensation in patients who survived at first decompensation (*N*=146)

Variable	Univariable analysis		Multivariable analysis	
	*P*	HR (95% CI)	*P*	HR (95% CI)
Recompensation	<0.001	0.24 (0.13 - 0.44)	<0.001	0.25 (0.13 - 0.48)
MELD score (per point)*	0.019	1.03 (1.01 - 1.06)	0.198	0.98 (0.94 - 1.01)
PT-INR (per unit)*	<0.001	2.54 (1.83 - 3.52)	<0.001	2.42 (1.48 - 3.95)
Albumin (per *g/dL*)*	0.001	0.49 (0.32 - 0.74)	0.071	0.68 (0.45 - 1.03)
Hemoglobin (per *g/dL*)*	0.001	0.88 (0.82 - 0.95)	0.029	0.92 (0.85 - 0.99)

*HR* Hazard ratio, *CI* Confidence interval, *MELD* Model for End-Stage Liver Disease, *PT-INR* Prothrombin time-international normalized ratio

* Baseline laboratory values at first decompensation

## Discussion

This prospective observational study highlights the impact of recompensation, as defined by the Baveno VII criteria, on the clinical course of patients with alcohol-related cirrhosis following their first decompensating event. Among the 184 patients, 45 (24.5%) achieved recompensation. Importantly, recompensation was associated with a 96% decrease in the risk of death or LT (adjusted HR = 0.04) and a 75% decrease in the risk of further decompensation (adjusted HR = 0.25).

Hepatic recompensation has recently been proposed as a novel stage of cirrhosis in the Baveno VII consensus. It is defined by the resolution of the primary etiology of liver disease, improvement in liver function, and absence of cirrhosis-related complications. Following the establishment of the Baveno VII criteria, recompensation rates ranging from 19% to 56% have been reported in Chinese patients with decompensated cirrhosis who achieved hepatitis B viral suppression [10–12, 15]. In Indian and European populations with decompensated cirrhosis who achieved hepatitis C virus eradication, the recompensation rates ranged from 25% to 63% [13, 14]. Delisting for clinical improvement, based on criteria besides the Baveno VII definition, has been reported in 9–16% of patients with alcoholic decompensated cirrhosis [23, 24]. However, the clinical implications of recompensation specifically defined by the Baveno VII criteria for alcohol-related cirrhosis are poorly understood. To date, limited evidence exists beyond a single-center retrospective study by Hofer et al. [16]., which found that only 18% of 204 abstinent Austrian patients achieved recompensation. In that study, recompensation was associated with decreased liver-related mortality (adjusted HR, 0.09; 95% CI = 0.01–0.68) and was more likely in patients with lower Child-Pugh scores and higher mean arterial pressure.

A key strength of our study is its prospective design, focusing on patients with alcohol-related cirrhosis following their first decompensation. Approximately 40% of patients achieved alcohol abstinence and 25% achieved recompensation after the first decompensation event. In our cohort, 35% (63/184) of the patients developed ACLF and 20% (38/184) died during the initial hospitalization. Lower Child-Pugh score and MELD scores at first decompensation were independent factors associated with recompensation in our study. This finding is consistent with previous studies, which have also identified lower Child-Pugh or MELD scores as significant factors for recompensation [16, 23, 24].

Recompensation represents a partial reversal of the structural and functional abnormalities of cirrhosis following the removal of the primary etiology of the liver disease. According to the Baveno VII definition, abstinence constitutes the removal of the primary etiology of alcohol-related cirrhosis, which is associated with significant survival benefits in both early and advanced stages of the disease [5, 9]. Consistent with these previous findings, our study also observed that abstinence itself was strongly associated with improved survival and reduced further decompensation, reinforcing its role as the fundamental driver of recompensation. The underlying pathophysiology of decompensation includes: (1) clinically significant portal hypertension; (2) hepatic fibrosis; (3) increased gut permeability; and (4) cirrhosis-associated immune dysfunction [25, 26]. Abstinence reduces systemic inflammation by improving gut permeability and bacterial translocation [27]. Furthermore, abstinence can reduce portal pressure, resulting in ascites resolution and decreased risk of recurrent VB [28]. In addition, alcohol abstinence improves cirrhosis-associated immune dysfunction in patients with liver disease [29].

A systematic review of 118 studies reported 1-year mortality rates of 60% in patients with decompensated cirrhosis, compared to just 2% in those with compensated cirrhosis [3]. In our study, achieving recompensation in patients with decompensated cirrhosis was associated with a significant survival benefit compared to those who remained decompensated (adjusted HR = 0.04). To accurately reflect long-term clinical outcomes, such as death, LT, and further decompensation, our analysis included 146 survivors, excluding those who died during the index hospitalization. Among those without recompensation, 60 of 101 (59.4%) patients experienced death or underwent LT, compared to just 1 of 45 (2.2%) patients who achieved recompensation. This suggests that patients with decompensated cirrhosis who achieve recompensation following abstinence may have survival rates comparable to those with typical compensated cirrhosis. Supporting this, Hui et al. reported that recompensation in hepatitis B-related cirrhosis was associated with survival rates similar to compensated cases [15].

Two previous European retrospective studies reported that approximately 60% of patients with cirrhosis who experienced a first decompensation experienced further decompensation based on the Baveno VII criteria [17, 30]. In our study, of the 146 patients who survived the first decompensating event, 95 (65%) experienced further decompensation. Among the patients who experienced further decompensation, 59 of 95 (62.1%) either died or underwent LT. Tonon et al. demonstrated that an etiological cure can prevent further decompensation [30]. Similarly, in our study, alcohol abstinence-driven recompensation was a significant factor in preventing further decompensation (adjusted HR = 0.25).

Nonetheless, this study has some limitations. First, alcohol abstinence was not assessed using a standardized questionnaire but was based on patient and caregiver reports during outpatient visits. Second, being a single-center study, generalizing the clinical course of patients with alcoholic cirrhosis after the first decompensation may be limited. Third, potential selection bias due to loss to follow-up within the first year cannot be entirely excluded, although this may partly reflect the referral of stabilized patients to local centers. Fourth, some patients died unexpectedly during the index hospitalization, which could have led to an inaccurate assessment of their previous decompensation history. Nonetheless, this is the first prospective study applying the Baveno VII criteria for recompensation in patients with alcohol-related cirrhosis. Finally, patients who achieved recompensation were included in the survival analysis regardless of subsequent alcohol relapse. Although this definition might overestimate the benefits of sustained recompensation, our subgroup analysis showed that patients who achieved recompensation, even with subsequent relapse, had better outcomes than those who never achieved it.

In conclusion, nearly one-quarter of the patients with decompensated alcohol-related cirrhosis achieved recompensation based on the Baveno VII criteria. Those who achieved recompensation through alcohol abstinence had significantly decreased risks of further decompensation and mortality. These findings support the need for multicenter prospective studies on the impact of recompensation in alcohol-related cirrhosis.

## Data Availability

The datasets generated and/or analyzed during the present study are not publicly available due to ethical and confidentiality reasons but are available from the corresponding author on reasonable request under the Gyeongsang National University Changwon Hospital Ethics Committee’s approval. The data that support the findings of this study are available on request to the corresponding author (Sang Soo Lee, Email: 3939lee@naver.com).

## Abbreviations

ALD: Alcohol-associated liver disease
LT: Liver transplantation
HE: Hepatic encephalopathy
VB: Variceal bleeding
MELD: Model for end-stage liver disease
ACLF: Acute-on-chronic liver failure
SBP: Spontaneous bacterial peritonitis
HRS-AKI: Hepatorenal syndrome–acute kidney injury
sHR: Subdistribution hazard ratio
CI: Confidence intervals
